# Establishing Requirements for Technology to Support Clinical Trial Retention: Systematic Scoping Review and Analysis Using Self-determination Theory

**DOI:** 10.2196/38159

**Published:** 2023-04-13

**Authors:** Eoin Gamble, Conor Linehan, Ciara Heavin

**Affiliations:** 1 School of Applied Psychology University College Cork Cork Ireland; 2 Lero Research Centre Cork Ireland; 3 Department of Business Information Systems Cork University Business School University College Cork Cork Ireland

**Keywords:** clinical trial, clinical research, retention strategies, participant retention, technology strategy, decentralized clinical trial, participant motivation, patient centric, engagement strategies, self-determination theory

## Abstract

**Background:**

Retaining participants in clinical trials is an established challenge. Currently, the industry is moving to a technology-mediated, decentralized model for running trials. The shift presents an opportunity for technology design to aid the participant experience and promote retention; however, there are many open questions regarding how this can be best supported. We advocate the adoption of a stronger theoretical position to improve the quality of design decisions for clinical trial technology to promote participant engagement.

**Objective:**

This study aimed to identify and analyze the types of retention strategies used in published clinical trials that successfully retain participants.

**Methods:**

A systematic scoping review was carried out on 6 electronic databases for articles published from 1990 to September 2020, namely CINAHL, The Cochrane Library, EBSCO, Embase, PsycINFO, and PubMed, using the concepts “retention,” “strategy,” “clinal trial,” and “clinical research.” This was followed by an analysis of the included articles through the lens of self-determination theory, an evidence-based theory of human motivation.

**Results:**

A total of 26 articles were included in this review. The motivational strategies identified in the clinical trials in our sample were categorized into 8 themes: autonomy; competence; relatedness; controlled motivation; branding, communication material, and marketing literature; contact, tracking, and scheduling methods and data collection; convenience to contribute to data collection; and organizational competence. The trials used a wide range of motivational strategies. Notably, the trials often relied on controlled motivation interventions and underused strategies to support intrinsic motivation. Moreover, traditional clinical trials relied heavily on human interaction and “relatedness” to support motivation and retention, which may cause problems in the move to technology-led decentralized trials. We found inconsistency in the data-reporting methods and that motivational theory–based approaches were not evident in strategy design.

**Conclusions:**

This study offers direction and a framework to guide digital technology design decisions for future decentralized clinical trials to enhance participant retention during clinical trials. This research defines previous clinical trial retention strategies in terms of participant motivation, identifies motivational strategies, and offers a rationale for selecting strategies that will improve retention. It emphasizes the benefits of using theoretical frameworks to analyze strategic approaches and aid decision-making to improve the quality of technology design decisions.

## Introduction

### Trial Retention and the Changing Landscape

Once a participant is enrolled in a clinical trial, retaining the participant and keeping them engaged and participating in the trial protocol is essential to the conduct of a valid and reliable trial [1] and to avoid the costs associated with patient withdrawals [2]. Clinical trial researchers and practitioners must implement strategies for supporting patient retention in a trial [3]. However, there is a surprising lack of research that seeks to understand, from both theoretical and practical perspectives, how to best retain participants once they are enrolled in a trial [4].

Two recent developments in the conduct of clinical trials have motivated this study. First, clinical trial researchers and practitioners have begun using digital tools as part of their trial experience [5-7], permitting improved patient-clinician communication to take place remotely [8]. Technology such as apps and wearables are often championed as a way to improve the overall patient experience during clinical trials. Such technologies present both opportunities and challenges to the clinical trial process and must be carefully designed to meet the desired outcomes. Second, there has been a move toward a remote decentralized clinical trial (DCT) model. For the most part, physical visits to a clinical center are eliminated, data capture takes place using mobile devices, and web-based participant-reported outcomes and interactions between trial staff and patients predominantly use technologies such as telemedicine [9]. However, there remains an open question regarding the strategies, features, and functions that potential trial technologies should contain to best support participant retention. To answer this question, we reviewed evidence from existing research to identify, describe, and then analyze what strategies practitioners currently use to motivate participants or patients retained in clinical trials.

### Background

In discussing the support of participant retention in clinical trials, we refer to the strategies and tactics used by trial designers and trial staff to keep patients enrolled and from withdrawing or “dropping out” of a clinical trial [3,10]. The retention of participants can be a major challenge, as over time, a participant’s motivation to remain in a study may decrease [11]. Strategies for addressing retention are used regularly with varying success rates, and retention costs are consistently high across all phases of trials [12]**.** Research to identify effective retention strategies has increased in recent years [13], but there is a lack of evidence for the effectiveness of individual strategies [14] and a need for more focus on retention-specific research [4].

Previous research has identified a range of motivations for participating in clinical trials. For example, many participants become involved owing to the altruistic desire to help others with the disease or condition in question [15]. Other participants hope to attain health benefits, gain access to new treatments, improve their treatment, gather new data about the disease, access laboratory testing, or take part because of trust in the physician [16,17]. Additional factors include the perceived benefits to society, the amount of care and attention received while enrolled in a study, research interest [18], their personal values [19], and financial benefits or monetary compensation received [17,18,20]. Despite these initial motivators to enlist in a trial, poor patient retention is frequently encountered during the conduct of trials.

Research suggests that the average patient attrition or dropout rate for a clinical trial is 30% [21], but dropout rates can range from 5% to 70% [22]. A variety of reasons are cited for patients withdrawing from trials, including life and study demands, logistics, lack of motivation, and overall commitment [23]. The timing of follow-up and scheduling contacts can also affect patients remaining in a trial [24], as can the complexities encountered during treatment [25]. Demographic characteristics such as age, gender, lower income, education, and literacy can predict both enrollment and patient attrition rates [26]. Factors such as culture, community practices, political outlook, and geography [22] are cited as influential factors. Attrition and noncompliance are attributed to the accessibility of health care information, consumer empowerment, and mistrust of research, all of which affect trial validity [19]. Other factors affecting patient participation during a trial process include unrelated illness, side effects, forgetting, and competing external stressors [27]. Personal, emotional, and psychosocial factors are cited as predictors of patient dropouts [28].

Owing to the problems identified with retaining participants in clinical trials, trial practitioners have developed practices related to both the design and implementation of trials that are intended to improve participant retention. Previous systematic reviews assessed the retention approaches in health care and clinical settings. For example, Robinson et al [29] set out to describe the range of retention strategies implemented in health care research, classifying their findings into 12 themes: community involvement, study identity, study personnel, study description, contact and scheduling methods, reminders, visit characteristics, benefits of study, financial incentives, reimbursement, nonfinancial incentives, and special tracking methods. Research showed that studies had a median of 17 strategies across a median of 6 themes. The most frequently reported strategies dealt with the themes of participant contact, scheduling, and minimizing patient burden. Robinson et al [30] published an updated review in 2015. The findings highlighted that the use of a larger number of retention strategies appeared to result in the retention of more participants. The study also highlighted the inability to identify what retention strategies were the most effective and the need for further research to evaluate the effectiveness of different strategies.

Several other relevant reviews were conducted, including the 2014 Cochrane review by Brueton et al [3], which aimed to quantify the effect of retention strategies used in 38 trials. Six types of strategies were evaluated: incentives, communication strategies, new questionnaire format, participant case management, behavioral and methodological interventions, and monetary incentives for questionnaire responses. There are several other reviews, including reviews examining recruitment and retention strategies in mental health trials [31], reviews identifying the characteristics of participants that might predict retention in trials involving children [32], and a review analyzing the use of patient incentivization to improve retention rates [33].

Existing research has identified that using a larger number of retention strategies appeared to retain more study participants. However, existing research does not provide a robust explicit rationale for why this is the case [30]. Owing to the shift to technology-led decentralized trials, we suggest that there is a need to explore participant retention in the context of both technology design and DCT. We advocate the adoption of a stronger theoretical position is required to improve the quality of technology-design decisions regarding participant motivation and retention.

### Supporting Participant Motivation Through Study Design

Several psychological theories exist that could prove beneficial in understanding how to guide participant behavior through trial design. These theories include the theory of planned behavior [34], health belief model [35], goal-setting theory for motivation [36], and transtheoretical model [37]. We suggest that the self-determination theory (SDT) provides a particularly useful lens through which to evaluate the retention strategies currently used in trials. The SDT is particularly applicable to the clinical setting because of the exploration of autonomy and autonomous self-regulation as core concerns and considerations of autonomy as an ethical mandate for patients to partake in medical research [38]. The SDT is a framework commonly used for guiding the design [39] and evaluation of digital health tools [40]. The SDT has been shown to be relevant and useful in studies involving clinical trial research and in health care settings [38,41,42]. The SDT is appropriate for understanding engagement and behavior changes related to digital experiences and technology design [39].

### Self-determination Theory

The SDT identifies the 3 basic psychological needs of competence, autonomy, and relatedness, and meeting these basic needs has been consistently shown to be associated with effective, motivated performance [43]. All 3 needs should be met to stimulate intrinsic autonomous human motivation, optimize performance, and regulate individuals’ behaviors [44]. The 3 basic psychological needs defined by the SDT are described in the following sections [39,43].

- Autonomy (acting in accordance with one’s goals and values): Autonomy refers to a sense of willingness and acting with a sense of volition and motivation in accordance with a person’s personal goals and values, which connects autonomy with meaning and purpose.
- Competence (feeling able and effective): Competence refers to the perception of being capable and effective. Optimal challenges, positive feedback, and opportunities for learning have been shown to enhance a sense of competence.
- Relatedness (feeling connected and a sense of belonging to others): Relatedness is defined as a sense of belonging and connectedness, with its core consisting of an individual’s feeling of closeness to and connection with others.

This is in contrast with controlled motivation, in which one’s behavior is a function of external contingencies, such as control, coercion, and obligation. In controlled motivation, rewards or punishments are used as motivation, and people engage as they believe that that is what is expected of them [45]. Controlled motivation involves compliance with pressure, and autonomous motivation involves behaving with a sense of volition, agency, and choice [46]. The consequences of controlled motivation are internal apprehension and pressure with lower performance and motivation [47]. Autonomous and controlled motivation lead to different outcomes, with conditions supportive of autonomy and competence facilitating growth tendencies and conditions that control behavior damaging those tendencies [44].

- Controlled motivation refers to approaches in which one’s behavior is a function of external contingencies of reward and punishment, and actions such as coercion and obligation are associated with this motivation [48].

Deci and Ryan [49] suggest that there are 2 main types of motivation: intrinsic and extrinsic motivations. Intrinsic motivation is behavior driven by internal motivators and rewards. This construct describes the natural inclination toward fulfillment, growth, enthusiasm, and satisfaction inherently arising from engaging in a behavior. Intrinsic motivation requires supportive conditions to thrive, and nonsupportive environments can disrupt this motivation. By contrast, extrinsic motivation refers to behavior driven by imposed conditions, external motivators, or offers of rewards for performance in an activity and can vary considerably in autonomy [50]. Both intrinsic and extrinsic motivations represent intentional behavior but vary in their relative autonomy. Intrinsic motivation is in many ways superior to extrinsic motivation, as an extrinsically motivated action tends to dissipate when given external controls are withdrawn [51]. The quality of experience and performance can vary based on whether one is acting for intrinsic or extrinsic reasons [50].

### This Study

This study built upon and added to previous reviews on patient retention in clinical trials. Similar to previous studies [30], we first identified and described the strategies used in trials that successfully retained participants. This review followed the methodological framework for systematic scoping reviews proposed by Arksey and O’Malley [52]. Subsequently, and novel to this review, we performed a theory-based analysis of the identified retention strategies through a modern theory of motivation, the SDT [43]. Using the SDT as a theoretical lens helped us examine the strategies to understand the motivational approaches used for patient retention in clinical trials. In addition, we reviewed the selected articles to establish whether the authors of existing studies applied a formal or named theoretical approach to influence and inform the development of the strategic approaches used to retain participants. Overall, our methodology enabled us to identify strategies, opportunities, and knowledge gaps and make recommendations for further research.

## Methods

### Identifying Relevant Studies

The search strategy targeted peer-reviewed published articles whose primary focus was on retention strategies and used the concepts of “retention,” “strategy,” “clinal trial,” and “clinical research.” Initial search was conducted on 6 electronic databases: CINAHL, The Cochrane Library, EBSCO, Embase, PsycINFO, and PubMed (see Multimedia Appendix 1). These databases were selected, as they were considered the most likely to contain the type of studies we were pursuing. Searches were conducted once in June and again in September 2020. The search strategy was limited to journal articles published from 1990 to 2020. The start date of 1990 was chosen because the authors concluded that 1990 and the years thereafter would have seen the availability and use of technology for running trials and incorporation of a variety of contemporary strategies for patient retention in clinical trials. Articles were considered for review only if they were published in the English language.

### Study Selection

Study selection was an iterative process. The process involved searching the literature, followed by screening to aid in the reduction of papers for inclusion (see Multimedia Appendix 2). Studies that were considered relevant were included if they conducted quantitative or qualitative research and were peer-reviewed publications. To be eligible for inclusion, papers needed to explicitly mention “clinical trials” or “clinical research” and “patient retention strategies” in the paper title, introduction, or keywords. Articles were required to describe the retention strategies used to retain enrolled patients in a clinical trial or evaluate those retention strategies’ effects or effectiveness. We included quantitative studies if they had an ≥80% retention rate, and the trial took place for a minimum of 6 months. Qualitative studies were included if they discussed retention strategies used in a clinical trial. The exclusion criteria for articles included not explicitly mentioning retention strategies in clinical trials or if the interventions used were not explicitly described in the article. An article was not eligible for consideration if the trial population was vulnerable or high-risk participants in areas such as substance abuse and addiction, mental health trials, patients with psychological issues, or children. We excluded articles if the trial had <80% retention rate or if the research lasted <6 months. In addition, systematic reviews were not included in our research.

**Figure 1:**
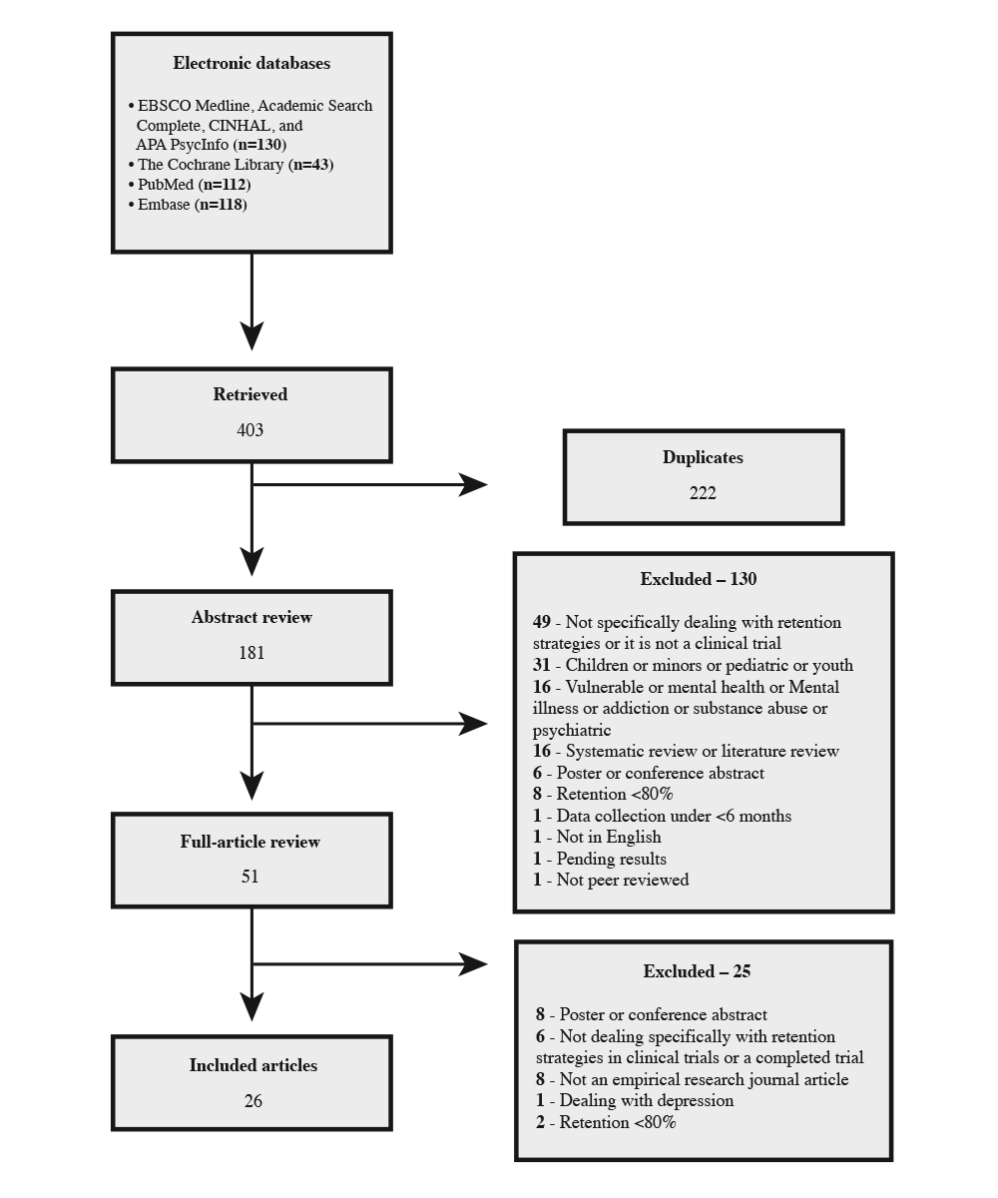
PRISMA (Preferred Reporting Items for Systematic Reviews and Meta-Analyses) flow diagram.

We retrieved a total of 403 relevant articles for further analysis. After removing duplicates, there were 44.9% (181/403) potentially relevant papers for the initial title and abstract screening. During this screening, if it was not possible to exclude an article, we obtained the full-text version and assessed eligibility based on it. Studies that did not meet the inclusion criteria were excluded. In total, 28.2% (51/181) of studies were included in the full article review following this comprehensive search strategy and screening process. Of these 51 studies, 25 (49%) were omitted: 8 (32%) studies were excluded because they were poster or conference abstracts, 6 (24%) studies were rejected because they did not explicitly deal with retention strategies in clinical trials, 8 (32%) studies were excluded because they were not empirical research studies, 2 (8%) studies had <80% retention rate, and 1 (4%) study considered vulnerable or high-risk participants. This review left 51% (26/51) of articles for inclusion. Refer to the PRISMA (Preferred Reporting Items for Systematic Reviews and Meta-Analyses) flow diagram in Figure 1.

### Charting the Data—Data Extraction

The next stage involved extracting information from the included qualitative and quantitative research studies. From each article, we recorded pertinent data (Textbox 1). We focused on relevant information with the aim of understanding the types of trials being conducted. The authors identified a list of retention strategies considered relevant to the participants enrolled and used during the clinical trial. Bias and potential errors were reduced by 2 reviewers analyzing the information during the data extraction process. Data from each article meeting the inclusion criteria were extracted by EG, who independently screened the literature search for relevant results at both phase 1 (titles and abstracts) and phase 2 (full-text articles). Then, CL independently assessed the studies for eligibility and accuracy.

**Extracted pertinent data.**

**Quantitative papers**

- Paper title
- Authors and publication date
- Retention results at 6 months, 9 months, 12 months, or 2 years
- Therapeutic area or type of trial conducted
- Trial population

**Qualitative papers**

- Paper name
- Authors and publication date
- Therapeutic area type of trial conducted
- Qualitative method
- Trial population

### Analysis Methods

We developed a mixed inductive and deductive method for analyzing and interpreting the retention strategies in our sample based on a thematic analysis process [53]. First, initial codes were generated through open coding and applied to the data. Specifically, this process involved the researcher labeling each retention strategy with a descriptive code (see Multimedia Appendix 3). Second, the open-coded retention strategies were deductively sorted into a set of theoretically important, predefined themes based on the SDT (refer to Textbox 2 for a list of these themes). This process allowed us to visualize the types of motivational strategies used by the studies in our sample according to a theoretically relevant perspective.

**Definitions of the higher-order themes that were used to deductively classify open-coded data.**

- Autonomy
  Interventions that create conditions that enable participants to take ownership of their actions and show willingness and volition concerning their behavior
- Competence
  Interventions that create conditions that enable participants to feel able and effective; experiencing opportunities and support such as positive feedback, education, training; and learning opportunities
- Relatedness
  Interventions that create conditions that enable participants to feel connected to others, offer a sense of belonging, or assist an individual’s feeling of closeness to and connection with others
- Controlled motivation
  Interventions in which behavior is prompted by the external contingencies of reward, coercion, and obligation
- Branding, communication material, and marketing literature
  Interventions using branding, communication material, and marketing literature to communicate with, promote the study to, and engage with patients during the trial process
- Contact, tracking, and scheduling methods and data collection
  Interventions and approaches that aid with the scheduling of patients to attend trial clinics and that aid in the tracking of patients during the trial and methods that aid in the collection of data from patients during the trial process
- Convenience to participate to collect data
  Interventions used to bring convenience to participants to enable data collection
- Organizational competence
  Operational management approaches, organization, skills, behaviors, and competencies to support organizational performance

Two reviewers independently coded the data to reduce the possibility of error and reach consensus. This evidence was summarized and presented for review.

## Results

### Overview

This review identified 26 studies that reported retention strategies in clinical trials. Of the 26 studies, 16 (62%) were quantitative (Table 1), and 10 (38%) were qualitative (Table 2). The quantitative studies sampled were conducted between 2004 and 2020. The maximum participant retention rate in the studies identified was 97.8%. The duration of the trials whose retention rates were recorded ranged from 6 months to 2 years. A mix of populations was identified in the 16 quantitative studies. Of these 16 studies, 4 (25%) studies focused on low-income groups, and the remaining 12 (75%) studies had various study populations. Regarding the types of trials, HIV prevention trials (n=3) and intervention studies (n=3) were the most common types of trials, followed by cancer (n=2) and diabetes trials (n=2).

A variety of study populations were enlisted to the qualitative research studies selected, and the participants were predominantly a mix of roles from site and study teams involved in conducting clinical trials. Table 2 highlights a variety of types of trials and study populations. The remaining studies focused on an assortment of trials. The 10 qualitative studies identified were conducted between 2005 and 2020. The most used method for gathering data was interview techniques (3/10, 30%), followed by surveys (2/10, 20%), a combination of surveys and interviews (2/10, 20%), focus groups (2/10, 20%), and workshops (1/10, 10%).

Table 3 shows the articles associated with the respective higher-order themes based on the strategies observed and displays whether theory influenced strategy design. In addition to the 4 themes taken directly from the SDT, 4 higher-order themes were generated during the analytical process. These additional higher-order themes were used to classify the data reviewed as follows: (1) branding, communication material, and marketing literature; (2) contact, tracking, and scheduling methods and data collection; (3) convenience to participate to collect data; and (4) organizational competence.

**Figure 2:**
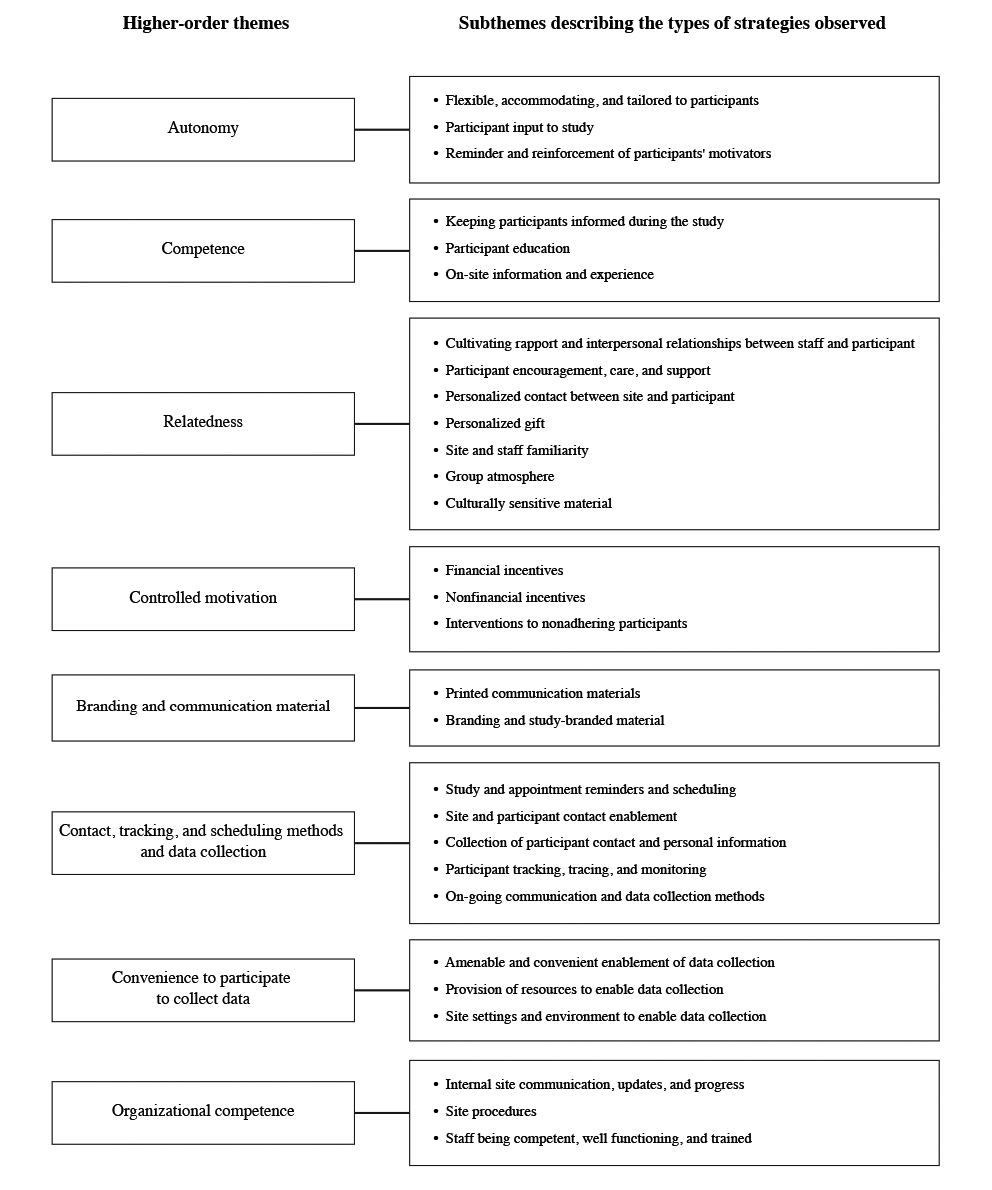
Higher-order themes and abstracted subthemes describing the types of strategies observed within each theme.

The coded strategies extracted were further inductively organized into subthemes that describe the types of strategies observed within each higher-order theme (Figure 2). The supporting subthemes and the respective retention strategies are summarized and presented for each theme in Figure 2.

**Table 1:** The quantitative papers identified with the various percentages of participant retention achieved, therapeutic area, and study population.

Paper	Authors and publication date	Retention at 6 months	Retention at 9 months	Retention at 12 months	Retention at 2 years	Therapeutic area and type of trial conducted	Trial population
A Collaborative Approach to the Recruitment and Retention of Minority Patients With Diabetes in Rural Community Health Centers	Davis et al [54], 2009	90.9%	—^a^	82.4%	—	Telemedicine-based diabetes self-management intervention	Minority adults aged ≥35 years with type 2 diabetes
A Review of Strategies Used to Retain Participants in Clinical Research During an Infectious Disease Outbreak: the PREVAIL I Ebola Vaccine Trial Experience	Browne et al [55], 2018	—	—	97.8%	—	Vaccine trial follow-up	Individuals aged 18 to 90 (median 30) years
Effective Recruitment and Retention Strategies for Older Members of Rural Minorities	Burns et al [56], 2008	94%	91%	—	—	Type 2 diabetes	Rural African American women aged >55 years
Engaging African American Breast Cancer Survivors in an Intervention Trial: Culture, Responsiveness and Community	Germino et al [57], 2013	—	—	87% at 11 months	—	Intervention trial	Caucasian and African American survivors of breast cancer
Impact of Financial Reimbursement on Retention Rates in Military Clinical Trial Research: a Natural Experiment Within a Multi-site Randomized Effectiveness Trial With Active Duty Service Members	Novak et al [58], 2019	—	95%	—	—	Collaborative care study	US active duty service members
Lessons Learned for Recruitment and Retention of Low-Income African Americans	Taani et al [59], 2020	83.1%	—	—	—	Hypertension self-management	African Americans with low income
Maximizing Participant Retention in a Phase 2B HIV Prevention Trial in Kampala, Uganda: the MTN-003 (VOICE) Study	Wynne et al [60], 2018	94%	95%	92%	—	HIV prevention	Women with a high risk for HIV
Novel Strategies Implemented to Ensure High Participant Retention Rates in a Community Based HIV Prevention Effectiveness Trial in South Africa and Zimbabwe	Gappoo et al [61], 2009	—	—	—	94%, 93%, and 89% across 3 sites	HIV prevention trial	Females who are sexually active and HIV negative
Patient-Centered Recruitment and Retention for a Randomized Controlled Study	Chhatre et al [62], 2018	—	—	—	Between 74% and 83% across 3 sites	Prostate cancer	Patients with localized prostate cancer
Recruiting and Retaining Low-Income, Multiethnic Women Into Randomized Controlled Trials: Successful Strategies and Staffing	Barnett et al [63], 2012	94%	—	—	—	Increase breastfeeding	Women from low-income, minority populations
Recruitment and Retention of Latinos in a Primary Care-Based Physical Activity and Diet Trial: the Resources for Health Study	Eakin et al [64], 2007	81%	—	—	—	RCT^a^ of a physical activity and dietary intervention	Predominantly Latino patients with low-income in a primary health care clinic
Retention Strategies and Factors Associated With Missed Visits Among Low Income Women at Increased Risk of HIV Acquisition in the US (HPTN 064)	Haley et al [65], 2014	93%	—	94%	—	HIV prevention trials	Women aged 18 to 44 years in zip codes with high poverty and HIV prevalence
Strategies to Improve Recruitment and Retention of Older Stroke Survivors to a Randomized Clinical Exercise Trial	Taylor-Piliae et al [66], 2014	81%	—	—	—	Exercise RCTs among survivors of stroke	Community-dwelling men and women who were aged ≥50 years and had stroke at least 3 months (range 53 months to 10 years) before
Strategies to Recruit and Retain Older Filipino–American Immigrants for a Cancer Screening Study	Maxwell et al [67], 2005	—	—	88%	76%	Cancer prevention, screening, and treatment trials	Female Filipino–American immigrants aged >40 years
Successful Recruitment and Retention Strategies for a Randomized Weight Management Trial for People With Diabetes Living in Rural, Medically Underserved Counties of South Carolina: the POWER Study	Parra-Medina et al [68], 2004	79%	—	81.5%	—	Weight management programs and usual care	Adults aged ≥45 years with diabetes, overweight, and those living in rural, medically underserved communities
Successful Strategies for Practice-Based Recruitment of Racial and Ethnic Minority Pregnant Women in a Randomized Controlled Trial: the IDEAS for a Healthy Baby Study	Goff et al [69], 2016	—	—	97.3% at 18 months	—	RCT of an intervention	Women belonging to low-income, racial and ethnic minority populations

^a^Not available.

^b^RCT: randomized controlled trial.

**Table 2:** The qualitative papers identified, therapeutic area, study participants, and trial population.

Paper	Authors and publication date	Therapeutic area and type of trial conducted	Study participants	Qualitative method	Trial population
Best Practice Guidance for the Use of Strategies to Improve Retention in Randomised Trials: Results From Two Consensus Development Workshops	Brueton et al [70], 2017	—^a^	UK clinical trials units, statisticians, clinicians, RCT^b^ coordinators, research scientists, research assistants, and data managers associated with RCTs participated	Workshop	—
Community Health Worker Perspectives on Recruitment and Retention of Recent Immigrant Women in a Randomized Clinical Trial	Choi et al [71], 2016	Randomized trial to promote mammogram and Pap tests	Community health workers	Focus groups	Korean Americans
Culturally Competent Strategies for Recruitment and Retention of African American Populations into Clinical Trials	Otado et al [72], 2015	Mixed studies	25 study coordinators	Survey or interview	Individuals with diverse cultural backgrounds, with the majority being African Americans
Evaluating and Improving Recruitment and Retention in an mHealth Clinical Trial: an Example of Iterating Methods During a Trial	Pfammatter et al [73], 2017	mHealth^c^ intervention to preserve and promote CVH^d^	Target population	Survey	College freshmen
Participant Retention Practices in Longitudinal Clinical Research Studies With High Retention Rates	Abshire et al [13], 2017	Longitudinal studies—mixed	Study team	Survey and in-depth, semistructured interviews	—
Recruitment, Recruitment, Recruitment’—the Need for More Focus on Retention: a Qualitative Study of Five Trials	Daykin et al [2], 2018	Randomized trials—mixed	Trial team members—chief investigators, trial managers, nurses, and research administrators	Semistructured interviews	—
Strategies for Retaining Study Participants in Behavioral Intervention Trials: Retention Experiences of the NIH Behavior Change Consortium	Coday et al [1], 2005	Behavioral intervention trials—studies targeted toward disease prevention through behavior change	15 university-based sites	Focus groups	Mixed
Use of Strategies to Improve Retention in Primary Care Randomized Trials: a Qualitative Study with In-depth Interviews	Brueton et al [74], 2014	Primary care randomized trials	29 UK primary care chief and principal investigators, trial managers, and research nurses	In-depth interviews	—
Identifying Research Priorities for Effective Retention Strategies in Clinical Trials	Kearney et al [14], 2017	—	Chief investigators or clinical trial units	Survey	—
Recruitment and Retention of Older Adults in Assisted Living Facilities to a Clinical Trial Using Technology for Falls Prevention: a Qualitative Case Study of Barriers and Facilitators	Meekes et al [75], 2020	Physical function	Scheme managers, therapists, and researchers	Semistructured interviews	Older adults living in assisted living facilities

^a^Not available.

^b^RCT: randomized controlled trial.

^c^mHealth: mobile health.

^d^CVH: cardiovascular health.

**Table 3:** Selected articles with the identified higher-order themes based on self-determination theory and analysis of theory in articles.

Paper	Branding, communication material, and marketing literature	Contact, tracking, and scheduling methods and data collection	Convenience to participate to collect data	Organizational competence	Controlled motivation	Autonomy	Competence	Relatedness	Theory mentioned
Abshire et al [13], 2017	✓	✓	✓	✓	✓	✓	✓	✓	
Barnett et al [63], 2012	✓	✓	✓		✓				
Browne et al [55], 2018		✓						✓	
Brueton et al [74], 2014	✓	✓	✓		✓	✓		✓	
Brueton et al [70], 2017	✓	✓			✓				
Burns et al [56], 2008		✓	✓		✓	✓		✓	
Chhatre et al [62], 2018		✓		✓	✓			✓	
Choi et al [71], 2016			✓	✓	✓			✓	
Coday et al [1], 2005	✓	✓	✓	✓	✓	✓	✓	✓	
Davis et al [54], 2009		✓	✓		✓			✓	
Daykin et al [2], 2018	✓	✓	✓	✓		✓	✓	✓	
Eakin et al [64], 2007		✓	✓			✓			
Gappoo et al [61], 2009		✓	✓		✓	✓	✓	✓	
Germino et al [57], 2013			✓		✓	✓	✓	✓	
Goff et al [69], 2016		✓		✓	✓	✓			
Haley et al [65], 2014	✓	✓	✓	✓	✓			✓	
Kearney et al [14], 2017		✓		✓					
Maxwell et al [67], 2005		✓			✓		✓	✓	
Meekes et al [75], 2020	✓						✓	✓	
Novak et al [58], 2019					✓				
Otado et al [72], 2015		✓	✓		✓	✓		✓	
Parra-Medina et al [68], 2004		✓			✓	✓		✓	
Pfammatter et al [73], 2017		✓			✓			✓	
Taani et al [59], 2020		✓		✓	✓		✓	✓	
Taylor-Piliae et al [66], 2014	✓	✓	✓		✓	✓		✓	
Wynne et al [60], 2018		✓		✓					

### Autonomy

This theme described interventions to enable participants to self-endorse and take ownership of their actions, offering flexibility and empowering participants’ willingness and volition concerning their behavior. The retention strategies coded under “Autonomy” were inductively grouped into 3 subthemes.

#### Flexibility, Accommodating, and Tailored to Participant

Studies used a range of strategies that attempted to improve participants’ autonomous experience by tailoring elements of the study experience, offering flexibility, and accommodating participants during the trial. The strategies observed included consideration of the participants’ circumstances, such as scheduling trial-related calls at a convenient time [57]. Staff made accommodations according to personal situations [68] and provided home follow-ups where necessary [74]. Staff anticipated and were responsive to participants’ needs, creating flexible protocols [69], and negotiated with participants regarding the amount of data they would collect [1]. Flexible appointments were offered [4], including after hours and weekends [72], and participants with difficulties attending clinics or who “no-showed” numerous times were accommodated [64]. Participants were given a “break” from calls, and messages were not left on every call attempt [1]. Tactics included data collectors offering flexibility to participants [57], staff behaving in a respectful manner [72], and site staff troubleshooting challenges encountered by participants [66]. Participants were consulted on the best time to call, data collection windows were extended, tests were modified and rescheduled, and the frequency and number of contacts of participants were reconsidered if the burden became substantial [1]. Staff encouraged participants to bring items from home to make themselves comfortable and adapted their approaches to individual participants’ situations [13].

#### Participant Input to Study

Some studies offered participants the opportunity to offer input directly to the study approach. A range of methods was observed, including involving community members in study planning and implementation [56], listening to participants’ concerns regarding the study and personal issues, and being sensitive to participants’ needs [72]. After visit, staff inquired about participants’ well-being, feelings about the studies, and possible concerns [72]. Clinics provided suggestion boxes, and signage was used, encouraging participants to notify the coordinator if waiting times exceeded 10 minutes [61].

#### Reminder and Reinforcement of Participants’ Motivators

During the trials, staff engaged participants and reminded them of the benefits of involvement and of the participants’ personal drivers to partake in the trial. This approach was used to remind and reinforce the personal drivers and independent commitment made by participants to partake in the trial. Instances included staff discussing the benefits and highlighting the advantages accrued from taking part in the study [13,68]. Participants were sent letters from their Doctor of Medicine, and staff discussed motivations, benefits, and reasons for joining the study, reminding participants of their commitment and encouraging follow-through while emphasizing the importance of helping others [1].

### Competence

#### Overview

This theme describes the observed strategies that aimed to enable the participants to feel able and effective. Strategies included offering support such as positive feedback, education, training, and learning. The retention strategies coded under “Competence” were inductively grouped into 3 subthemes.

#### Keeping Participants Informed During the Study

Strategies used in a number of studies attempted to keep participants informed about the trial, specifically about trial activities and progress, giving participants knowledge and understanding of aspects related to the studies. The methods observed included sending participants personalized letters with an update on study activities and selected study findings [67]. Staff motivated participants by supporting them to feel confident and safe, feedback was provided on performance, and participants shared positive experiences [75].

#### Participant Education

This subtheme describes interventions intended to educate and improve the participants’ understanding of trial procedures, disease management, and health. The strategies observed included delivering intervention material in person and staff reviewing and training participants [57]. Advice and education were provided by staff to participants during the trial [75], addressing participants’ health literacy [59], offering clear and transparent data collection procedures [4], and highlighting the need for complete data [1]. Studies hosted educational discussion forums and group discussions related to disease management, encouraging participants to invite the study team to provide education and conduct testing at family gatherings and reunions [13].

#### On-site Information and Experience

The research found strategies in studies wherein staff endeavored to create an on-site experience that was understandable to participants and encouraged a comforting experience, enabling greater comprehension and comfort at the site. Participants were encouraged to bring a blanket or pillow from home to make themselves comfortable [13]. Signage positioned in the clinic waiting areas and bathrooms communicated with and gave direction to participants; a liaison officer was deployed for waiting participants to provide guidance and share information about the expected waiting periods [61].

### Relatedness

The strategies observed in this theme aimed to enable participants to feel connected, offer a sense of belonging, and assist an individual’s closeness to others. The retention strategies coded under “Relatedness” were inductively grouped into 6 subthemes.

#### Cultivating Rapport and Interpersonal Relationships Between Staff and Participant

Trials used interventions conducive to cultivating interpersonal relationships, building rapport, and fostering connections and affiliations between participants and study staff. Methods to enhance retention included the moral compass of individual trial team members [4] and staff alleviating participant fear and doubts [55]. Trial teams aimed to maintain good relationships [71] and build trusting [56], respectful, and confidential relationships with participants [61]. Staff tried to maintain a good rapport [72,74], keep contact using social media [65], be charismatic [66], and build continued interpersonal relationships with participants [4]. The staff adopted a nonpatronizing approach in which showing traits of being approachable and supportive were regarded as necessary [75].

#### Participant Encouragement, Care, and Support

As for this subtheme, the studies used several interventions, including behaving in a manner that offered encouragement and displayed care and support to participants. Some examples include the researchers showing characteristics of being approachable and supervising and providing support to participants with the aim of making them feel confident and safe [75]. Staff used informal interpersonal relationship strategies by showing a caring nature [4]. Staff gave participants genuine attention and care and fulfilled their needs using community resources, such as medication [71]. When dealing with participants, staff listened, helped with problem-solving, and used verbal thanks [1]. Staff made accommodations, communicated with enthusiasm, showed concern for personal situations [68], and offered personalized attention and encouragement [66]. The people in participants’ contact list, including relatives, friends, significant others, physicians, and health clinics, were called and asked to encourage participants and promote the benefits of continued participation [1]. Participants with strong personalities taking part in the study and peers sharing positive experiences and cheering and clapping for each other were used to create an environment of encouragement [75]. A newsletter consisting of testimonials of former participants was circulated, and staff offered encouragement by reminding participants of the importance of helping others and the national scope of the group [1].

#### Personalized Contact Between Site and Participant

Studies highlighted staff exercising strategies such as the personalization of contacts made and interactions and touchpoints with participants. Examples include sending individualized [57] and personalized letters with an update on study activities [67]. These included personalized notes and cards maintaining ongoing individualized contact and engagement, such as those given on holidays and birthdays and those conveying thank yous and greetings [1,13,57,59,62,65]. Cards were sent from the group or staff to check on the status of a participant who became ill, in the event of a family death or illness [1], and to offer condolences [13]. Staff would also follow-up if participants became ill during the trial, even if the participants’ illness had nothing to do with the trial [4]. Staff sent email greetings for birthdays, holidays, and important events [73]. Electronic systems generated automated birthday greetings and reminder telephone call lists for research staff [68]. To build rapport, staff made monthly calls [65], made touch-base calls, or sent touch-base emails between scheduled visits [1]. After visit, calls were made to inquire about participants, gauge their feelings, and hear concerns regarding the study [72].

#### Personalized Gift

Studies used personalized approaches such as mailing personalized gifts to participants every 2 months [57]. Examples of such gifts included gifts of pictures of the participants with their child, peers, or staff [1]. In another study, calendars featuring artwork created by the participants or their grandchildren were sent to trial participants in mail correspondences [13].

#### Site and Staff Familiarity

The strategies observed emphasized participants’ familiarity with the study staff and trial site. Examples of these strategies included conducting the trial research at community health centers with which participants might be familiar [54]. Staff wore name tags [61] and uniforms [75]. Participants were allocated to 1 team member as a primary contact [13], and the same staff members were deployed throughout the follow-up period, aiding the development of strong staff-participant relationships [13,56]. Partnership with local, well-known, and trusted clinics and clinicians increased the credibility of the study and aided retention [59].

#### Group Atmosphere

The study sites sought to create an atmosphere within the study group that was positive for participants. An example of this is staff maintaining a comfortable atmosphere in the study group during sessions [75].

#### Culturally Sensitive Materials

Sites used culturally appropriate language and study symbols in study material to ensure that it would be acceptable to rural, older African American participants [56].

### Controlled Motivation

This theme describes interventions using strategies that attempt to influence participant behavior through external contingencies such as reward and coercion. The retention strategies coded under “Controlled motivation” were then inductively grouped into 3 subthemes.

#### Financial Incentives

Trials used financial incentives to encourage retention and engagement. Participants were compensated for their continued involvement in the trial. The financial incentives observed in our sample of studies included gift cards [54,66], gift certificates [1], reimbursements [58], and cash [13,56,59,69,74]. Compensation was given to participants upon completion of various stages of the trial [1,57,63,65,67,68] and for providing questionnaire responses [70]. Contributions to participants’ churches were also made [71].

#### Nonfinancial Incentives

Several nonfinancial incentives or small gifts were also used to encourage and reward participants for continued participation in the trial. Nonfinancial [70] incentives included T-shirts, tote bags, pens, magnets, key rings, mugs, and certificates of completion [54,74]. Community resources, such as additional medication and referrals, were used to fulfill the needs of participants [71]. Parking validation, tickets, and lunch [72] were the other incentives used. Award systems [61], lottery systems [61,67], coupon raffles [56], gifts [57], retention events such as spa days and luncheons [65], and annual events at entertainment venues [13] were also used. The opportunity to receive medical procedures and consultation was provided [1]. Lifestyle-related incentives were provided [1] to encourage participants, and a booklet and supporting DVD and CD were used to promote physical activities [66].

#### Interventions for Nonadhering Participants

This subtheme describes interventions delivered by trial staff upon recognizing a participant’s lack of adherence to the study protocol or in the event that a participant becomes nonresponsive or noncompliant or misses visits. For example, in many studies, staff responded immediately to missed appointments by visiting the participant on the same or subsequent day to reschedule the appointments [61] or contacting them by telephone [62,68], by letter [67], or using several approaches, including text, email, phone call, postcard, and via social media, depending on participant responses [73]. For interviews, participants who were uncontactable received personalized letters and emails [63]. Other approaches included staff emphasizing the need for complete data, making expectations clear to participants at initial contact, or begging and pleading with the participants [1]. Coordinators and site directors visited participants that were deemed “difficult” [61].

### Branding and Communication Material

The first theme describes interventions that use branding, communication material, and marketing literature to communicate with, promote to, and engage participants during the trial process. The retention strategies coded under “Branding and communication material” were inductively grouped into 2 subthemes.

#### Printed Communication Material

Printed communication material was used in many studies in our sample. The observed strategies included the use of letters and traditional postal services for routine communication with participants [70,74]. Studies sent newsletters to participants [1,4,13,66] to keep them informed about general trial news [74]. Study brochures provided participants with relevant contact information [63], and studies used professionally printed questionnaires [4].

#### Branding and Study-Branded Materials

Many studies emphasized the importance of including study-related branding in communication material. For example, studies provided participants with study logo–branded materials, such as magnets [63], branded gospel music tapes, audiotapes, potholders [1], key chains, T-shirts [65], and branded artwork calendars [13]. The wearing of uniforms with a logo was also considered important [75]. Studies conducted by universities and government-funded agencies used their brand associations [75].

### Contact, Tracking, and Scheduling Methods

This theme describes strategies, interventions, and approaches that aid with scheduling participants to attend trial clinics and tracking participants and methods that aid with data collection during the trial process. Strategies coded under “Contact, tracking, and scheduling methods” were inductively grouped into 5 subthemes.

#### Study and Appointment Reminders and Scheduling

This subtheme describes appointments and scheduling approaches used and considered valuable strategies to retain participants, including study and visit reminders [13,55,61]. Participants received appointment cards for follow-up visits [61], postcard reminders [63], and visit reminders, and calendars showing the sequence of visits for the study duration were issued at enrollment [60]. Staff engaged participants with reminders of their upcoming appointments and clinic visits [55] via telephone calls [54,72], postcards [65,72], or reminder letters [1,61,65]. The timing of the approach was important, and before or leading up to a visit or a scheduled appointment, a phone call was triggered [1,54,60,61]. The staff gave in-person and postal reminders during the week preceding the scheduled visit [61] and sent letters about the study and upcoming visits at regular intervals [13]. At the visits, participants received an in-person reminder of the upcoming interview [63]. The retention approach also included general contact and scheduling [13] and multiple rescheduling of visits for participants [1]. As a scheduling technique, research staff disclosed incentives to participants when scheduling visits by telephone [68]. The research team attempted to contact participants 3 times by phone, texts, or emails, and if unsuccessful, a letter was sent [59]. Scheduling and calendar software, visit window reports, and prompts were used by site staff [1].

#### Site and Participant Contact Enablement

The strategies in this subtheme offered participants multiple ways to enable participants to contact sites. Examples included sites providing participants with study-labeled material with the study’s contact information and business card containing phone numbers [63], selecting appropriate individuals with responsibility for participant contact, and allocating a study’s primary point of contact to a group of participants [13]. Sites provided participants with toll-free numbers to enable communication [54,56,65]. Participants provided additional contact information, including those of family members or friends, to allow for multiple means of contact [54].

#### Collection of Participants’ Contact and Personal Information

The strategies observed in this subtheme included the collection of participant contact and personal information. The approaches incorporated participants providing their own contact and location information and the contact information of additional individuals to allow multiple means of communication [4,13,60,61,63]. Alternate contact lists were established [66], and reference for contacting these individuals and permission to mention the trial name were noted [61]. The contact information was reviewed with the participants and updated if necessary [13,54,63]. Staff checked the clinic database and participant charts for updated numbers to replace incorrect details [64] and updated the locator forms at each visit [61]. Studies requested participants to provide full family names and nicknames by which they were known in their communities and to describe the area in which they lived, allowing for more effective follow-up in rural areas [55].

#### Participant Tracking, Tracing, and Monitoring

This subtheme describes interventions for participant tracking, tracing, and monitoring that were used as strategies for supporting retention endeavors and included procedures, methods, and systems for tracking participants and monitoring data return. Tracking methods were used to inform retention efforts and facilitate communication among team members [13]. Sites collected detailed participant locator information [61], and the attendance and progress of participants were monitored [14,61,66,68]. Participants’ progress was tracked through the study [69] using the tracking system [68] and spreadsheets or databases [13]. To maintain a good tracking system of participants including phone availability, notes and sites recorded call attempts [1,61]. To ensure a comprehensive participant search process, staff executed a checklist of techniques [13] and generated a list of participants who attended follow-up visits [60]. Staff checked rosters, directory listings, transfers, addresses, or phone changes [1]. Searches were also conducted using web-based prison and incarceration records and jail databases [13,65]. Death registries and the local newspaper obituary were also used to locate participants [65]. Staff searched the medical records of participants who were typically more difficult to contact for any upcoming clinic visits [63]. Staff visited participants’ homes and used personal delivery of reminders and court documents when locating participants [13]. Other methods used to locate participants included the use of phone numbers, email, texting, social media, and internet searches [13], and GPS was used to identify homes in areas that are difficult to access [61]. Letters were sent to ask participants to inform sites about changes in address or telephone numbers [67]. Lists of participants who missed visits were created and distributed to community outreach workers [60], and staff were alert and responsive to potential signs of participant dropout [1]. Monthly reports identified potential problem sites, staff meetings were held to address the issues, and community outreach workers discussed challenging cases. They used locator information to trace participants and understand the reason for nonattendance [60]. Browne et al [55] discussed how trials in Liberia hired trackers from local communities who were familiar with the local culture, geographic area, community leaders, residents, and population. Local teams embraced cultural norms and had local knowledge, and trial participants were assigned to a tracker who spoke with family and friends to locate mobile participants. Owing to the participants’ lack of access to mobile phones, the phone number of one of the participants was collected and used as a central contact number to cover a group of participants. The phone number of a community store was also used, and store employees would locate the participants.

#### Ongoing Communication and Data Collection Methods

The research found that several trials implemented strategies facilitating ongoing communication and the collection of data from participants. Examples included employing several staff members to collect data, manage retention [13], and make frequent follow-up contact [56]. Team members, including the principal investigator [1], visited participants, collected information [55,61], and used the telephone to contact participants [59,74]. Staff recontacted participants at intervals for short interviews [67], and ongoing communication took place through various means such as text, email, letter, and phone [59,70,74]. Staff repeated attempts to recontact participants [64], and they gave participants a break from contact and did not leave messages at every call attempt [1]. Nurses reinforced clinic messaging in the various communities in which participants resided [55], and plain English was used during data collection [70].

### Convenience for Data Collection

#### Overview

The strategies observed in this theme centered on convenience to contribute to data collection. Staff used strategies to make the trial more conducive to data collection. The retention strategies coded under “Convenience to participate in collecting data” were inductively grouped into 3 subthemes.

#### Amenable and Convenient Enablement of Data Collection

Several interventions were adopted to optimize retention when collecting data by offering flexibility to participants and attempting to reduce the data collection burden on them, accommodating the participants’ lifestyle, and making the trial procedures more convenient for the participant when providing data. Examples included the flexibility of data collectors [57] and streamlining appointments [74] to accommodate participants. To facilitate participation, staff were accommodating when scheduling face-to-face interviews and visits and scheduled visits around other appointments [1]. Participants tolerance for data collection activities was addressed by shortening visits, collecting “primary” measures first before collecting other data, modifying tests, and rescheduling appointments if participants were injured or ill [1]. Proposing home visits [1,65,74] and conducting interventions in participants’ homes [56,61,64] were considered convenient and helped alleviate transportation difficulties. Sites performed assessments and interventions at the same location [66], implemented short patient visits [65], and made additional trips to collect samples [57]. Some trials used community-based visit locations for convenience [65]. For participants who faced partner violence, arranging a safe meeting place was proposed [13]. Strategies included being flexible by accommodating participants’ schedules, after-hour and weekend appointments [72], and Saturday and evening clinics [61]. Sites offered appointments outside traditional workdays [65], extending hours [1] and widening time windows of 4 to 6 weeks to achieve more success in reaching participants [64]. Some studies offered participants the option to return questionnaires or return to sites for follow-up and found more convenient ways to collect participant data, such as via the web or telephone [74], and if participants moved location, they were offered alternate options such as phone or mail visits [1]. Scheduling calls per participants’ convenience [57] and asking whether an alternate number was better to call on [1] was deemed meaningful for participant retention. Conducting calls on weekday evenings and weekends [63,64] and modifying the number of contacts if perceived as too burdensome to a participant were other strategies used [1]. Factors used to optimize retention included new questionnaire formats, convenience in data collection such as web-based collection, the reduction of time participants spent in follow-up activities, and the reduction of types of data collected (eg, long questionnaires and biomedical specimens) [74]. The decrease in outcome measure burden was considered effective [4], and for working or hard-to-reach participants, an award system for completed visits was implemented to allow them to jump the queue [61].

#### Provision of Resources to Enable Data Collection

Strategies used by sites to collect data from participants included arranging and providing resources to participants before, during, and after gathering data. Sites provided transportation to participants to get them to the trial sites [13,54,61,65]. Carpools were arranged [1], and sites reimbursed participants for transportation or gasoline [1,13]. Sites also provided metro or bus cards [65] to facilitate transport and enable participants to visit trial sites. Free car parking was arranged [4] and made available and accessible [1,66], and expenses were reimbursed [13] to the participant. Sites provided participants with toll-free numbers [54,56] and toll-free phone lines [65] to facilitate site contact. A clinic attendance letter was provided to present to a school or employer [61]. For participants with responsibilities for children, childcare was organized [13,65], and help was offered to arrange babysitting [1]. Participants were provided with postage-paid return envelopes to mail back questionnaires [1]. Troubleshooting the challenges participants were encountering [66] and providing resources such as medication to fulfill participants’ additional needs were other strategies that were followed [71]. Sites also provided a community resource guide with lists of local social services and food pantries, and woman well-care was handed out [65].

#### Site Settings and Environment to Enable Data Collection

The subtheme showed that sites endeavored to create an environment that was familiar and calming for the participants to enable comfortable surroundings throughout the trial. The research was conducted at established locations with which participants were familiar, such as community health centers [54]. Participants were encouraged to bring items from their homes to make themselves comfortable [13]. Family movies were played in waiting areas, and client liaison officers were employed to work with participants [61]. Meals, coffee, and snacks were provided at the trial sites after testing [13].

### Organizational Competence

This theme describes strategies centered on operational management approaches, organization, procedures, skills, and competencies to aid sites’ optimal performance. The retention strategies coded under “Organizational competence” were inductively grouped into 4 subthemes.

#### Internal Site Communication, Updates, and Progress

As part of this subtheme, there were several strategies that centered on-site communication efforts, progress, and updates for sites. These included regular team conference calls, retention workshops throughout the study [65], provision of study updates, and letters of progress reports [62]. Research highlighted the importance of good site relationships, regular contact [14], and internal communication among members of research teams [13,59]. Methods included study teams examining tracking methods, reviewing the latest retention rates, discussing ideas, strategizing, providing support, making changes, and adopting revised retention strategies [13]. Monthly reports were used to help identify potential problem sites, and lists of participants who missed their visits were also created and distributed [60].

#### Site Procedures

Studies emphasized procedures, processes, goal setting, and monitoring as part of retention efforts. Examples included sites using clear and transparent data collection procedures [4] and staff attending or being present at most sessions [69]. A systematic checklist of techniques for participant searches was used, including spreadsheets and databases, enabling the monitoring, evaluation, adjustment, or innovation of retention strategies [13]. Sites set daily participant contact goals, implemented friendly competition [13], and used incentives [4] as means to engage sites. Procedures such as recording call attempts to discern participants’ availability and taking notes on whether a message was left were followed [1].

#### Staff Being Competent, Well Functioning, and Trained

The subtheme included strategies focusing on the enhancement of the competencies, capabilities, and experience of site staff and training-aided trial retention strategies. Examples included study and site training [65], improving the phone skills of callers [1], and providing training to staff [69] at points such as initiation and triggered training [14]. Providing intensive training and support on study protocols, including retention techniques, focused on providing staff education on empathy, sensitivity, and mock interviews in preparation for the team engaging with participants [13]. The experience of trial team members [4]; staff bonding [1]; effective, organized, and persistent trial team functioning [13]; the competency of health workers [71]; and the ability to anticipate and respond to participants’ needs [69] were all deemed necessary to aid retention. The accessibility to research staff, including physicians’ ongoing involvement in the study and staff staying up to date to answer any arising participant questions [62], also aided retention. Team development approaches [59] and developing collaboration with clinic staff [69] were also fundamental to these strategies. Consultation with other sites regarding best practices [65], the use of team approaches, and employing several staff members were also deemed important to retain participants. Some studies employed a full-time staff member dedicated to implementing retention strategies and optimizing participant follow-up; the team offered strategic support, including adapting or developing new approaches to overcome retention barriers [13].

### Theoretical Framework

Upon review, none of the identified papers explicitly mentioned the use of theory to guide trial design and implementation (Table 3). On the basis our analysis, a formal theoretical approach to understanding and supporting human motivation is lacking in the reviewed literature. Conversely, if a theoretical framework was used to form strategies or influence strategic direction, the authors of the identified papers did not share this knowledge.

## Discussion

### Principal Findings

This systematic review of participant retention in clinical trials was conducted to identify and describe the strategies currently being used in trials that successfully retain participants. We analyzed the retention strategies observed in our sample of papers through a modern theory of motivation called the SDT to reveal patterns in the strategies. Specifically, we classified each strategy based on components of the SDT that speak to different types of human needs satisfaction (eg, the need for autonomy or the need for relatedness). We used this to reveal a picture of the diverse motivational strategies implemented to retain participants in clinical trials. From the 26 articles included, we identified examples of interventions that create conditions for all 3 SDT motivational themes, namely autonomy, relatedness, and competence, and strategies that fit into the controlled motivation themes. Most studies had a mix of several different strategic retention themes, highlighting the diversity of strategies used as part of trials (Table 3). Relatedness was the most prominent theme and had the largest number of associated subthemes. A substantial number of studies also showed the use of strategies based on controlled motivation. Of the strategies used that did not fit into our motivational model, contact, tracking, and scheduling methods and data collection yielded the most results. Although they did not fit into our participant motivation framework, they play an important role when conducting a trial. None of the papers reviewed explicitly mentioned the use of a theoretical framework guiding their retention strategy design decisions or as part of the formulation of strategies to retain participants. Theory consideration may have occurred during this process, but it was not evident in this collection of papers.

### Use of Controlled Motivation

A substantial proportion of the reviewed papers used motivation strategies that, upon analysis through our framework, can be characterized as controlled motivation. These strategies were used to influence participant behavior through external contingencies such as reward and coercion—financial and nonfinancial incentives and interventions such as reminders used for uncontactable and nonadhering participants. Traditionally, these strategies were considered integral for promoting participant engagement and clinical trial retention. However, it is important to note that according to the SDT, these controlled motivation strategies can have a detrimental effect and create conditions and influence participants in ways not conducive to remaining motivated [50]. The outcomes may include damaging motivational tendencies, lower performance, and possible demotivation to continue participating in the trial. Practitioners and designers may need to re-examine their reliance on these strategies because of the potential negative repercussions and potential to undermine retention. If these strategies are essential, they could be augmented with more autonomous and relatedness- and competence-based motivational approaches.

### Importance of Relatedness and Human Interaction

A recurring theme in patient retention literature is the importance of interpersonal relationships for engaging and motivating participants [56,71], and our study supported this point. Furthermore, our findings emphasized the reliance of existing trials on creating conditions for relatedness and the importance of human interaction during those trials. These strategies relied on staff and participants interacting with each other; staff’s responsiveness to, encouragement of, and communication with participants; and staff showing their human, respectful, genuine, caring, and considerate side. The behaviors and personal characteristics of staff and rapport and positive interactions with participants are integral to strategic approaches, and this illustrated the importance of rapport and interrelationships during the trial. The findings reinforced the importance of relatedness to participant motivation and retention. Replicating these interactions, strategies, and delivery of methodologies through technology will be challenging and potentially impractical for the new landscape of DCT. There are challenges and opportunities for designing technology and creating digital strategies and conditions that can enhance relatedness for participants as trials continue to move to the decentralized model.

### Role of Theory in Informing Patient Retention Strategies

The reviewed articles showed a surprising lack of research seeking to understand participant motivation from theoretical and practical perspectives (Table 3). A number of strategies used in the trials studied encompassed characteristics that are likely to create the conditions for motivation. However, the reviewed papers did not explicitly report the use of a theoretical framework of motivation when making decisions about which strategies should be used. In terms of theoretical motivation frameworks, there was little evidence that strategy was guided by a coherent understanding of human motivation, as several strategic approaches displayed characteristics that fit into the description of controlled motivation, which could, in turn, demotivate participants. We advocate using the theoretical framework of the SDT to guide the research and design of digital retention strategies. Using a theory-driven approach can aid in designing a digital strategy by assisting in the understanding of why and how an intervention works. Using a theoretical motivation framework as part of clinical trial technology retention strategies will help close the gap between current retention strategies and the design and implementation of digital retention strategies.

### Strategy Implementation

Past research has identified that using a larger number of retention strategies produces better retention results [30]. There is very little guidance on which strategies are appropriate for use in which context. Designers and researchers have just been encouraged to use a wide range of strategies. In our findings, we see that there is often reliance on trial staff and their experience to implement motivational strategies. It is difficult to imagine how we can replicate these important functions when trials move to decentralized, technology-facilitated models in which less human interaction is involved. When planning trials, we are not provided with actionable information other than using several different types of strategies, which is inefficient and difficult to justify for technology developers. Moving to the digital space will require a more measured and theory-driven approach to devising digital retention strategies. Doing so can allow the implementation and measurement of individualized, tailored strategies for participants.

### Intrinsic Participant Motivation

Many of the strategies reviewed focused on the operational and process approaches of sites; trial design; and contact, tracking, and data collection methods. All are interdependent elements for an organized trial experience for participants and are essential to ensure as seamless an experience as possible for the participant. Nevertheless, they do not contribute directly to participants’ intrinsic motivation or fit into the SDT model for motivation. We found a small sample of intrinsic motivation examples, such as staff reminding participants of their motivators, including benefits of involvement, and reminding them of original drivers to partake in the trial. To create more motivational experiences for participants, we suggest a more strategic focus on the participants’ personal motivators and delivering individually tailored strategies that emphasize these intrinsic motivators.

### Data Reported

The data-reporting approaches presented in the reviewed papers were often represented differently in the different studies observed. There were various reporting approaches to calculating the retention figures, and the interpretation of overall retention differed from one study to another. Owing to various interpretations, the variety of approaches made it challenging to represent and compare data regarding retention across trials. As we progress, this variety of approaches may obscure the ability to compare data to identify and evaluate effective retention strategies based on successful retention results. Standardization of reporting and a common approach to reporting retention results would be beneficial.

### Implications for Designing Technology

Applying the SDT framework in the design and evaluation of digital strategies and using the insights identified and organized in this paper will aid in defining the targeted strategic approaches. The insights and guidelines can be used for clinical trial participant technology design, offering a defined model to explain psychological approaches and provide actionable insights into digital retention strategies. This approach will aid the comprehension of the strategies, functions, features, and types of content that will assist improved engagement through technology experiences that support participant motivation by focusing on our digital experiences that concentrate on autonomy, relatedness, and competence. Using this approach and the clarity gained will allow us to advance patient digital retention strategies and create supportive conditions for improved methods for motivating participants. The methodology can enable us to recognize the strategies that might best serve to target the various psychological needs defined by the SDT to motivate and engage participants at multiple stages during their trial experience. The practice can clarify what kind of retention engagement we are trying to create and the reactions and types of motivation that might be elicited from each strategy. By using the SDT, it would provide designers with clarity and direction to tailor strategies that focus on the 3 basic psychological needs of participants defined by the SDT. Having clear goals regarding the variety of motivational retention strategies implemented will enable clear testing parameters to evaluate participants’ feedback and assess behavioral goals and effects on participants’ psychological needs for each strategy. Moreover, this will allow us to identify strategies that may not support the conditions needed for motivation. Our analysis can act as a source for implementing technology-based trial supports and behavior-influencing digital strategies with clear motivational and behavioral goals for digital technology.

### Limitations and Future Research

As with all qualitative analyses, the authors used their judgment when coding strategies by theme and subtheme, and we acknowledge that this approach allows room for error. Owing to our inclusion and exclusion criteria, this review featured strategies from quantitative papers with a threshold of ≥80% retention. This approach could have potentially omitted several strategies that may have benefited the review. The search approach used a limited number of keywords and omitted MeSH (Medical Subject Headings) terms because of the specific topic of interest, and this may have reduced the number of records extracted. The framework proposed by Arksey and O’Malley [52] was followed, but hand searching was not performed during the systematic review, as it was not considered essential to review every retention strategy. Our aim was to reveal the patterns of patient retention strategies more broadly. Future research will present opportunities for both academia and practice. The use of the SDT as a part of strategic interventions should be examined in future work, as we attempt to support both participants’ experiences and practitioners’ attempts to develop more engaging digital experiences. Opportunities are also presented to conduct research on digital engagement strategies that could create conditions for relatedness interventions to aid retention during trials.

### Conclusions

As technology advances, strategies and approaches used in digital tools for direct patient engagement will need to become more precise and more user centered to bridge the gap in retaining the 30% of patients who withdraw from trials. Retaining participants is a multifaceted phenomenon, with various personal, emotional, health, environmental, cultural, design, and contextual factors affecting retention during the trial process. The diversity of motives as to why a participant might join or drop out of a trial highlights the complexity of retaining patients in trials and the need to address participant motivation during the trial process. To develop the science of clinical trial retention in the context of digital technology, it is vital to improve research designs and methodologies centered on the digital space. This review underscores the importance of studying participant retention strategies through a theory of motivation. It enabled us to gain clarity on the previous retention strategic approaches used and define those strategies in terms of participant motivation. The research allowed us to identify strategies that fit into a motivational model and offer direction for designers. This examination is an early step in using the SDT framework to guide digital technology design and strategies for enhancing participant motivation during trials.

## Appendix

Multimedia Appendix 1 Database-specific search strategy material and search terms.

Multimedia Appendix 2 Search result articles and specific exclusion rationale.

Multimedia Appendix 3 Extracted segregated data by theme and sub theme.

## Abbreviations

DCT: decentralized clinical trial
MeSH: Medical Subject Headings
PRISMA: Preferred Reporting Items for Systematic Reviews and Meta-Analyses
SDT: self-determination theory
