# Supplementary material for: Establishing Requirements for Technology to Support Clinical Trial Retention: Systematic Scoping Review and Analysis Using Self-determination Theory
Source: J Med Internet Res. 2023 Apr 13;25:e38159. doi: 10.2196/38159 (PMC10141281; doi:10.2196/38159)
Supplement: Multimedia Appendix 1 [file jmir_v25i1e38159_app1.pdf]

Multimedia Appendix 1: **Database-specific search strategy and search terms**

The aim of the searches was to identify evidence for the questions being asked.

Database searches

The re-run searches took place on 29th of Sept 2020.

Published Dates

The search was conducted on articles published between January 1990 and September 2020.

Search Terms

"Clinical trial*" or "Clinical research" AND "Retention strateg*"

Main database searches

Sources searched for research papers

- **Ebsco**
  Academic Search Complete
  CINAHL Plus with Full Text
  MEDLINE
  APA PsycArticles
  APA PsycInfo
  Social Sciences Full Text (H.W. Wilson)
  SocINDEX with Full Text
- **PubMed**
  PubMed is the free U.S. National Library of Medicine's (NLM®) database of biomedical citations and abstracts.
- **Cochrane Library**
  The Cochrane Library is a collection of databases that contain high-quality, independent evidence to inform healthcare decision-making.
- **Embase**
  Embase is a biomedical and pharmacological database which gives you access to the most up-to-date information about medical and drug-related subjects.
